# Multiple Energy Transfer in Luminescence-Tunable Single-Phased Phosphor NaGdTiO_4_: Tm^3+^, Dy^3+^, Sm^3+^

**DOI:** 10.3390/nano10071249

**Published:** 2020-06-27

**Authors:** Jun Xiao, Cong Wang, Xin Min, Xiaowen Wu, Yangai Liu, Zhaohui Huang, Minghao Fang

**Affiliations:** Beijing Key Laboratory of Materials Utilization of Nonmetallic Minerals and Solid Wastes, National Laboratory of Mineral Materials, School of Materials Science and Technology, China University of Geosciences (Beijing), Beijing 100083, China; 2103190016@cugb.edu.cn (J.X.); 1930675@tongji.edu.cn (C.W.); xwwu@cugb.edu.cn (X.W.); liuyang@cugb.edu.cn (Y.L.); huang118@cugb.edu.cn (Z.H.); fmh@cugb.edu.cn (M.F.)

**Keywords:** phosphor, NaGdTiO_4_, energy transfer, luminescence, WLEDs

## Abstract

Advances in solid-state white-light-emitting diodes (WLEDs) necessitate the urgent development of highly efficient single-phase phosphors with tunable photoluminescence properties. Herein, the Tm^3+^, Dy^3+^, and Sm^3+^ ions are incorporated into the orthorhombic NaGdTiO_4_ (NGT) phosphors, resulting in phosphors that fulfill the aforementioned requirement. The emission spectrum of Tm^3+^ ions overlaps well with the adsorption spectra of both Dy^3+^ and Sm^3+^ ions. Under the excitation at 358 nm, the single-phase NaGdTiO_4_: Tm^3+^, Dy^3+^, Sm^3+^ phosphor exhibits tunable emission peaks in the blue, yellow, and red regions simultaneously, resulting in an intense white-light emission. The coexisting energy transfer behaviors from Tm^3+^ to Dy^3+^ and Sm^3+^ ions and the energy transfer from Dy^3+^ to Sm^3+^ ions are demonstrated to be responsible for this phenomenon. The phosphors with multiple energy transfers enable the development of single-phase white-light-emitting phosphors for phosphor-converted WLEDs.

## 1. Introduction

White-light-emitting diodes (WLEDs) are considered as the next generation of green lighting sources because of their low energy consumption, high output efficiency, and environmental friendliness, compared with traditional incandescent and fluorescent lamps [1,2,3,4]. At present, there are two methods are available for generating white light: the multichip combination method, in which red, green, and blue LED chips are used in combination, and the light conversion method, in which various phosphors are excited by blue, ultraviolet (UV), or near-UV LED chips [5,6]. The multichip combination method is limited by high costs and low performance. In the light conversion method, a yellow yttrium aluminum garnet phosphor powder such as (Y_1-a_Gd_a_)_3_(Al_1-b_Ga_b_)_5_O_12_:Ce^3+^ is excited by a blue LED chip [7,8,9]; the white-light is finally obtained when the blue and yellow light are combined, which is a more popular approach in high-efficiency commercial LEDs. However, the lack of a red-light component in the combined white-light results in a low color-rendering index, which is the main weakness of this approach for lighting [10,11]. This disadvantage appears to be solvable by combining red, blue, and green tricolor phosphors excited by UV or near-UV LED chips. However, energy reabsorption between various phosphors results in unsatisfactory luminous efficiency [12]. Therefore, the development of a single-phase white phosphor that is excited by UV and near-UV chips and that exhibits high luminous efficiency, high color stability, and good reproducibility remains a challenge.

The Dy^3+^ ion is commonly used as an activator for white-light-emitting phosphors because of its two emission peaks in the blue and yellow regions. The blue-light emission peak is located at 486 nm and corresponds to the ^4^F_9/2_→^6^H_15/2_ transition of Dy^3+^ ions, whereas the yellow-light emission at 579 nm is related to the ^4^F_9/2_→^6^H_13/2_ transition [13,14,15]. Both of these transitions are highly sensitive to the crystal-field environment. Therefore, the yellow- and blue-light emissions can be well regulated by manipulating the crystal-field environment of the Dy^3+^ ion, thus enabling white-light emission with a perfect color-rendering. However, because the blue-emission peak at 486 nm is not a pure blue emission, the co-doping of Tm^3+^ ions, which emit blue light under UV excitation, can lead to better white-light emission upon adjustment of the doping concentrations of the Tm^3+^ and Dy^3+^ ions [16,17]. However, the white light produced by the Tm^3+^/Dy^3+^ co-doped phosphor still lacks a red component; thus, the color-rendering index and color temperature are low, resulting in cool white-light emission. Among all the other rare-earth ions, Sm^3+^ can exhibit an orange-red-light emission under UV excitation [18], suggesting that it might be a suitable supplement for providing a red-light component to improve the color-rendering index and transferring the emission from the cool to the warm white-light region. Therefore, in the present work, Tm^3+^, Dy^3+^, and Sm^3+^ ions were co-doped into a NaGdTiO_4_ (NGT) matrix to obtain a single-phase white phosphor with improved luminescence properties.

**Figure 1:**
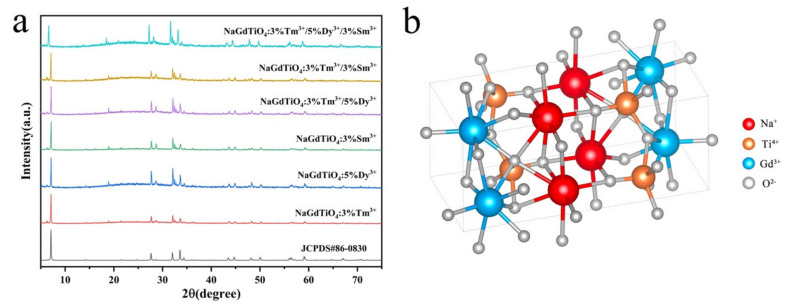
(**a**) XRD patterns of NaGdTiO_4_ (NGT): 3%Tm^3+^, NGT: 5%Dy^3+^, NGT: 3%Sm^3+^, NGT: 3%Tm^3+^/5%Dy^3+^, NGT: 3%Tm^3+^/3%Sm^3+^, and NGT: 3%Tm^3+^/5%Dy^3+^/3%Sm^3+^, and the Joint Committee on Powder Diffraction Standards (JCPDS) standard card for NaGdTiO_4_; (**b**) crystal structure of NaGdTiO_4_.

Among the matrix compounds used for phosphors, which include phosphates, tungstates, molybdates, and vanadates, titanates offer several advantages, including low cost, easy preparation, and good thermal stability [19,20,21,22,23,24]. NGT is a typical perovskite-structured titanate with a two-dimensional (2D) layered structure (Figure 1b). In this structure, the ionic radius of Gd^3+^ is similar to those of Tm^3+^, Dy^3+^, and Sm^3+^ ions, which theoretically enables the formation of a continuous solid solution without changing the original crystal structure [25,26]. In addition, NGT exhibits excellent chemical and thermal stability and strong absorption in the near-UV region [27,28]. Compared with the traditional inorganic phosphor materials, NGT has a relatively high critical concentration because of the limited energy transfer in the semi-2D sublattice [29]. Thus, we prepared the NGT-based phosphors doped with various activator ions (Tm^3+^, Dy^3+^, and Sm^3+^) via a conventional solid-state reaction and studied their luminescence properties, energy transfer behavior, and tunable emission properties in detail.

## 2. Material Synthesis and Characterization

The NGT: Tm^3+^, NGT: Dy^3+^, and NGT: Sm^3+^ single-doped phosphors; NGT: Tm^3+^/Dy^3+^, NGT: Tm^3+^/Sm^3+^ co-doped phosphors; and NGT: Tm^3+^/Dy^3+^/Sm^3+^ tri-doped phosphors were prepared via a high-temperature solid-state reaction method. High-purity TiO_2_, Gd_2_O_3_, Dy_2_O_3_, and Sm_2_O_3_ produced by Tianjin Guangfu Fine Research Institute (Tianjin, China); high-purity Tm_2_O_3_ produced by Shanghai Aladdin Biochemical Technology Co., Ltd. (Shanghai, China); and analytically pure Na_2_CO_3_ produced by Sinopharm Chemical Reagent Co., Ltd. (Shanghai, China) were used as raw materials.

First, appropriate amounts of the reactants were weighed according to the desired stoichiometric ratio, and an excess of Na_2_CO_3_ (30%) was added as a flux. The weighed compounds were transferred to a mortar and thoroughly mixed and ground for a few minutes. The resultant mixture was transferred to a crucible, which was subsequently placed in the center of a muffle furnace. The mixture was heated to 1000 °C at a rate of 10 °C/min and maintained at this temperature for 2 h under an air atmosphere. After naturally cooling to room temperature, the product was collected and thoroughly ground; the excess Na_2_CO_3_ in the product was removed by centrifugation with deionized water. After centrifugation, the product was placed in an oven and dried at 120 °C. After drying sufficiently, the sample was again ground for characterization.

Samples were first scanned using an X-ray powder diffractometer (DRU Advance, BRUKER, Billerica, MA, USA) over the 2θ range of 5–80° at a scan step width of 0.02° and a dwell time of 0.05 s per step. The phase behavior and purity of the samples were determined by comparing the XRD pattern of each sample to that reported in the standard card (JCPDS #86-0830). The morphology of the sample was observed using a Zeiss SUPRA-55 field-emission scanning electron microscope (Oberkochen, Germany). The elemental distribution of the sample area was qualitatively and quantitatively studied using energy spectra recorded with a field-emission scanning electron microscope (JSM-7001F, JEOL, Ltd., Tokyo, Japan). The photoluminescence excitation (PLE) spectra and photoluminescence (PL) emission spectra were recorded at room temperature using a Hitachi F-4600 fluorescence spectrometer (Hitachi, Japan). The excitation light source was a 150 W ozone-free Xe lamp; the scanning speed was 240 nm/min, and the photomultiplier tube voltage was 600 V. The PL quantum yields (QY) were recorded by a 10-inch integrating sphere (LMS-100, Labsphere Inc., North Sutton, NH, USA) with a multichannel CCD detector (USB QE Pro-65, Ocean Optics Inc., Edinburgh, UK). The decay curves and lifetimes of the samples were recorded using an Edinburgh FLS1000 fluorescence spectrometer (Edinburgh, UK).

## 3. Results and Discussion

### 3.1. Phase Composition and Morphologies

Figure 1a shows the XRD patterns of the as-prepared samples NGT: 3%Tm^3+^, NGT: 5%Dy^3+^, NGT: 3%Sm^3+^, NGT: 3%Tm^3+^/5%Dy^3+^, NGT: 3%Tm^3+^/3%Sm^3+^, and NGT: 3%Tm^3+^/5%Dy^3+^/3%Sm^3+^. All of the XRD patterns substantially match the standard card (JCPDS #86-0830) of NaGdTiO_4_. The results indicate that the phase compositions for both the single-doped and co-doped phosphors were stable, which means that the rare-earth dopants Tm^3+^, Dy^3+^, and Sm^3+^ did not change the crystal structure of the products. According to the crystal structure of NGT shown in Figure 1b, the ionic radius of Gd^3+^ (0.1053 nm) in coordination with oxygen atoms is similar to the radii of Tm^3+^ ion (0.0994 nm), Dy^3+^ ion (0.1027 nm), and Sm^3+^ ion (0.1079 nm) in the same coordination environment [30]. However, the charge of the Na^+^ ion and the radius of the Ti^4+^ ion vary dramatically from those of rare-earth ions. Thus, the Tm^3+^, Dy^3+^, and Sm^3+^ ions likely substitute Gd^3+^ ions, maintaining the stability of the NGT structure.

**Figure 2:**
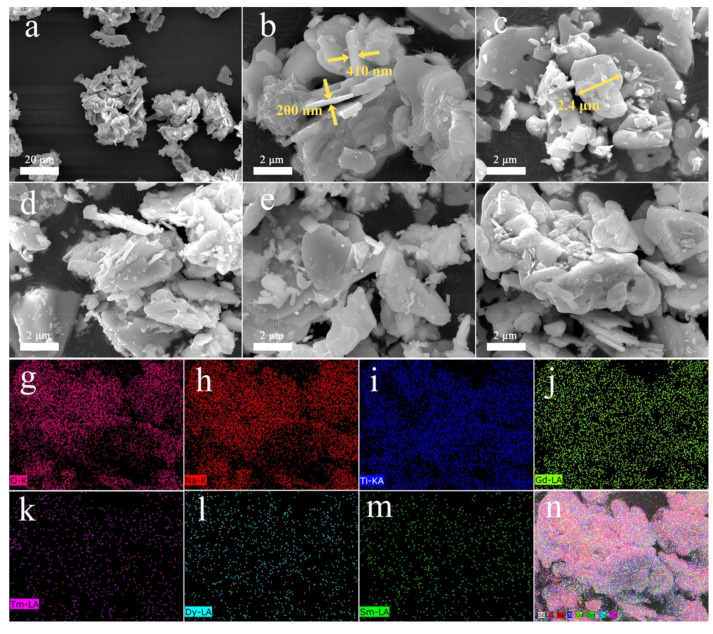
(**a**–**f**) SEM images of NGT: 3%Tm^3+^, NGT: 5%Dy^3+^, NGT: 3%Sm^3+^, NGT: 3%Tm^3+^/5%Dy^3+^, NGT: 3%Tm^3+^/3%Sm^3+^, and NGT: 3%Tm^3+^/5%Dy^3+^/3%Sm^3+^; (**g**–**n**) EDS mapping results of the NGT: 3%Tm^3+^/5%Dy^3+^/3%Sm^3+^.

The SEM images of the single-doped phosphors, co-doped phosphors, and tri-doped phosphor (Figure 2a–f) show that their morphologies are similar. All of the samples are composed of flakes with an average length of 1.5–2.5 μm and thickness of ~200–600 nm. To further confirm the doping of Tm^3+^, Dy^3+^, and Sm^3+^ ions into the NGT flakes, EDS mapping images were collected (Figure 2g–n). The results indicate that the Tm^3+^, Dy^3+^ and Sm^3+^ are uniformly distributed on the surface of the NGT: Tm^3+^/Dy^3+^/Sm^3+^ flakes, further confirming that these elements are effectively doped into NGT host.

### 3.2. PL Properties of NGT: Dy^3+^, NGT: Tm^3+^, and NGT: Sm^3+^

Figure S1a shows the PLE spectra of NGT: *y*%Dy^3+^ (*y* = 1, 3, 5, 7, 9) phosphors when monitoring at 579 nm. The phosphor with a doping amount of 3% exhibits the highest excitation intensity. The PLE spectra comprise a series of sharp excitation peaks in the region from 310 nm to 500 nm. These excitation peaks, centered at 326, 352, 366, 388, 427, 453, and 473 nm, are attributed to the transitions from ^6^H_15/2_ to ^4^M_17/2_, ^9^P_7/2_, ^4^P_5/2_, ^4^I_13/2_, ^4^G_11/2_, ^4^I_15/2_, and ^4^F_9/2_, respectively [14,15]. These excitation peaks are caused by the *f*-*f* transitions in the 4*f*^6^ shell of Dy^3+^ ions.

The PL spectra of the NGT: *y*%Dy^3+^ phosphors excited at an excitation wavelength of 352 nm are shown in Figure S1b. Two distinct characteristic emission peaks are observed at 486 nm and 579 nm, along with one weak emission peak at 670 nm. These emission peaks correspond to the ^4^F_9/2_→^6^H_15/2_, ^4^F_9/2_→^6^H_13/2_, and ^4^F_9/2_→^6^H_11/2_ transitions of Dy^3+^ ions, respectively [14,15]. Moreover, with increasing Dy^3+^ concentration from 1% to 7%, the luminescence intensity gradually increases, reaches a maximum at 3%, and then rapidly decreases with a further increase in concentration. The concentration quenching mainly arises from an increase in probability of nonradiative interaction with increasing distance between Dy^3+^ ions at higher concentrations.

To explore the main mechanism of concentration quenching of NGT: Dy^3+^ phosphors, the van Uitert equation (Equation (S1)) was used to fit the emission intensity at different concentrations [31]. In this equation, *Q* values of 3, 6, 8, and 10 represent exchange interactions, electric dipole-electric dipole interactions, electric dipole–electric quadrupole interactions, and electric quadrupole–electric quadrupole interactions, respectively. The emission intensity as a function of the concentration of Dy^3+^ ions is shown in Figure S1c. According to the fitted curves, the *Q* values of the yellow-light and blue-light emission are 5.89 and 6.13, respectively, both of which are very close to 6. This result indicates that the main mechanism of the concentration quenching of the NGT: Dy^3+^ phosphors is the electric dipole–electric dipole interaction. Furthermore, the color coordinates of the NGT: 3%Dy^3+^ phosphor were calculated to be (0.3504, 0.3783).

Figure S1d,e shows the PLE and PL spectra, respectively, of the NGT: *x*%Tm^3+^ (*x* = 1, 2, 3, 5, 7) phosphors. One strong blue-emission peak at 459 nm due to the ^1^D_2_→^3^F_4_ transition is obvious [32]. The emission intensity increases with increasing Tm^3+^ doping amount from 1%, reaches a maximum at 3% (Figure S1f), and then begins to decrease because of concentration quenching. The color coordinates of the NGT: 3%Tm^3+^ phosphor are calculated to be (0.1673, 0.0953), which fall within the blue region.

The PLE and PL spectra of the NGT: *z*%Sm^3+^ (*z* = 1, 2, 3, 4, 5) phosphors were also recorded (Figure S1g,h). Under excitation at 409 nm, the phosphors show distinct characteristic peaks at 568, 606, and 653 nm in their PL spectra, corresponding to the ^4^G_5/2_→^6^H_5/2_, ^4^G_5/2_→^6^H_7/2_, and ^4^G_5/2_→^6^H_9/2_ transitions of Sm^3+^, respectively [18]. Among these emission peaks, the one at 606 nm is much more intense than the others, resulting in a strong orange-red emission. As the Sm^3+^ concentration increases from 1% to 5%, the luminescence intensity first increases and then begins to decrease when the concentration is greater than 3% (Figure S1i), which is again attributed to concentration quenching. The calculated color coordinates of NGT: 3%Sm^3+^ are (0.5737, 0.4189), indicating that this phosphor can potentially be used as the red emitter for WLEDs.

**Figure 3:**
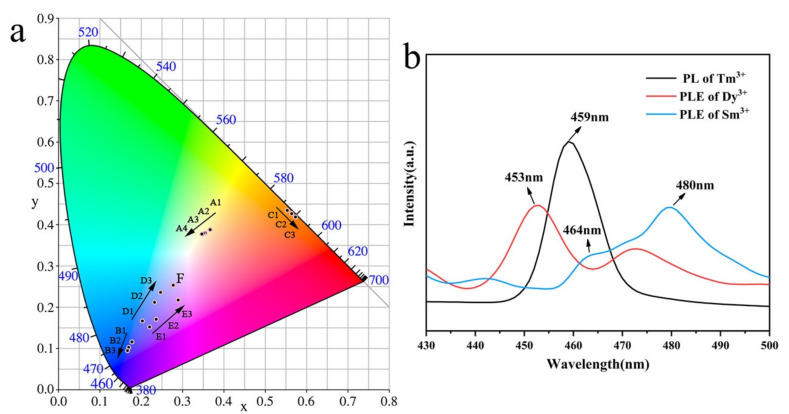
(**a**) The CIE chromaticity diagram of NGT: *x*%Tm^3+^, NGT: *y*%Dy^3+^, NGT: *z*%Sm^3+^, NGT: 3%Tm^3+^/*m*%Dy^3+^, NGT: 3%Tm^3+^/*n*%Sm^3+^, and NGT: 3%Tm^3+^/5%Dy^3+^/2%Sm^3+^ phosphors. Points A1–A4 represent NGT: *y*%Dy^3+^ (*y* = 1, 3, 7, 9) phosphors, points B1–B3 represent NGT: *x*%Tm^3+^ (*x* = 1, 2, 3) phosphors, points C1–C3 represent NGT: *z*%Sm^3+^ (*z* = 1, 2, 3) phosphors, points D1–D3 represent NGT: 3%Tm^3+^/*m*%Dy^3+^ (*m* = 1, 2, 3) phosphors, points E1–E3 represent NGT: 3%Tm^3+^/*n*%Sm^3+^ (*n* = 1, 2, 4) phosphors, and point F represents the NGT: 3%Tm^3+^/5%Dy^3+^/2%Sm^3+^ phosphors. (**b**) Overlap between the photoluminescence (PL) emission spectrum of the NGT: Tm^3+^ phosphor and the PL excitation spectra of the NGT: Dy^3+^ and NGT: Sm^3+^.

**Figure 4:**
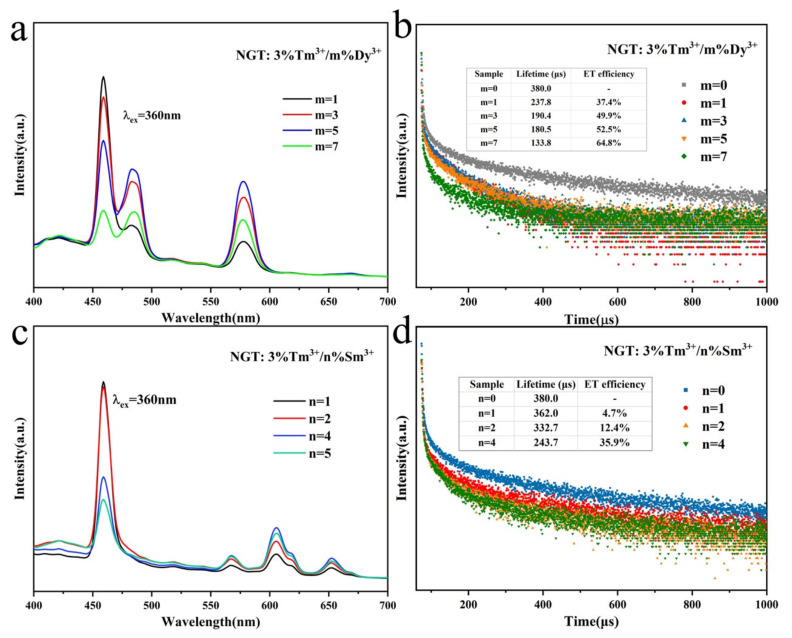
(**a**,**c**) PL emission spectra of NGT: 3%Tm^3+^/*m*%Dy^3+^ and NGT: 3%Tm^3+^/*n*%Sm^3+^ phosphors; (**b**,**d**) decay curves of NGT: 3%Tm^3+^/*m*%Dy^3+^ and NGT: 3%Tm^3+^/*n*%Sm^3+^ phosphors monitored at 459 nm and excited at 360 nm; the inset tables show their average lifetimes and energy transfer (ET) efficiencies.

As shown in Figure S1 and Figure 3b, the emission peak at 459 nm of NGT: Tm^3+^ overlaps well with the excitation peaks of both NGT: Dy^3+^ and NGT: Sm^3+^ phosphors in the absorption region from 440 to 480 nm, which may result in efficient energy transfer form Tm^3+^ to Dy^3+^ or Sm^3+^ ions [33]. Thus, we co-doped Dy^3+^ and Sm^3+^ ions with Tm^3+^ ions in an NGT host. First, the PL spectra of NGT: 3%Tm^3+^/*m*%Dy^3+^ (*m* = 1, 3, 5, 7) phosphors at an excitation wavelength of 360 nm were recorded (Figure 4a). These spectra have distinct characteristic emission peaks of Dy^3+^ and Tm^3+^ ions at 459, 483, and 578 nm, corresponding to the ^1^D_2_→^3^F_4_ transition of Tm^3+^ and the ^4^F_9/2_→^6^H_15/2_ and ^4^F_9/2_→^6^H_13/2_ transitions of Dy^3+^, respectively [17]. As the concentration of Dy^3+^ ions increases, the intensity of the emission peaks of Dy^3+^ gradually increases until the Dy^3+^ concentration reaches 5%. By contrast, the intensity of the emission peak corresponding to Tm^3+^ ions decreases with increasing concentration of Dy^3+^ ions, confirming the energy transfer behavior from Tm^3+^ ions to Dy^3+^ ions. As a result, the PL spectra and color coordinates can be tuned by controlling the doping concentration of Dy^3+^ ions (Figure 3a and Table S1).

All of the NGT: 3%Tm^3+^/*m*%Dy^3+^ phosphors exhibit cool white-light emission because of their lack of red emission. Thus, doping with rare-earth ions that can compensate for the emission in the red region, such as Sm^3+^ ions, is necessary. On the basis of this approach, we prepared NGT: 3%Tm^3+^/*n*%Sm^3+^ phosphors; the PL spectra at an excitation wavelength of 360 nm are presented in Figure 4c. Obvious characteristic emission peaks are observed at 459, 567, 606, and 652 nm, which are attributed to the ^1^D_2_→^3^F_4_ transition of Tm^3+^ and the ^4^G_5/2_→^6^H_5/2_, ^4^G_5/2_→^6^H_7/2_, and ^4^G_5/2_→^6^H_9/2_ transitions of Sm^3+^ ions, respectively [18]. When the Sm^3+^ concentration increases, the characteristic emission peak intensity of Tm^3+^ decreases, whereas the characteristic emission intensity of Sm^3+^ increases and reaches a maximum when the Sm^3+^ doping concentration is 4% because of the energy transfer between Tm^3+^ and Sm^3+^. Thus, the color coordinates are also tunable; those of the NGT: 3%Tm^3+^/4%Sm^3+^ phosphor are calculated to be (0.2891, 0.2171) because of the red-emission contribution of the Sm^3+^ ions.

To further confirm the energy transfer from the Tm^3+^ to Dy^3+^ and Sm^3+^ ions, we recorded the decay curves and average lifetimes of Tm^3+^ ions in the NGT: 3%Tm^3+^/*m*%Dy^3+^ and NGT: 3%Tm^3+^/*n*%Sm^3+^ phosphors (Figure 4b,d). All of the decay curves indicate that the emission intensities decrease with increasing decay time. With increasing concentrations of Dy^3+^ and Sm^3+^ ions, the emission intensity decays much more quickly. Furthermore, the decay curves were fitted through a double-exponential function based on Equations (S2) and (S3) [34]. The average lifetimes *τ* were calculated and are shown in the inset tables in Figure 4. The lifetimes decrease with increasing concentration of both Dy^3+^ and Sm^3+^ ions, implying that the diminished lifetimes mainly result from the increasing energy transfer from Tm^3+^ ions to Dy^3+^ and Sm^3+^ ions at high Dy^3+^ and Sm^3+^ doping concentrations.

Equation (S4) was used to calculated the energy transfer efficiencies (*η*) from the sensitizer Tm^3+^ to the activators Dy^3+^ and Sm^3+^ according to the intrinsic decay lifetime of the sensitizer with (*I_s_*) and without (*I*_0_) an activator [35]. The calculated *η* values are shown in the inset of Figure 4b,d, where the *η* values are observed to increase gradually with increasing activator dopant concentration. The *η* values also reached a relatively large value when the doping concentration was suitable for intense emission.

### 3.3. PL Properties and Energy Transfer Behavior of NGT: Tm^3+^/Dy^3+^/Sm^3+^

**Figure 5:**
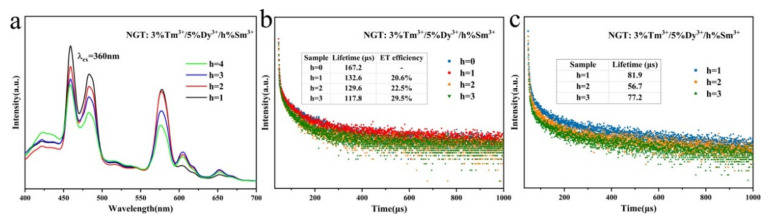
(**a**) PL emission spectra of NGT: 3%Tm^3+^/5%Dy^3+^/*z*%Sm^3+^ phosphors under the excitation of 360 nm. (**b**,**c**) The decay curves of NGT: 3%Tm^3+^/5%Dy^3+^/*z*%Sm^3+^ phosphors monitored at 459 and 487 nm, with excitation at 360 nm. The inset tables show the average lifetimes and energy transfer (ET) efficiencies.

To further improve the emission properties of the as-prepared phosphors, we co-doped the Sm^3+^ ion Tm^3+^ and Dy^3+^ ions in the NGT host. The emission spectra of NGT: 3%Tm^3+^/5%Dy^3+^/*h*%Sm^3+^ phosphors excited at an excitation wavelength at 360 nm are shown in Figure 5a. The characteristic emission peaks of Tm^3+^, Dy^3+^, and Sm^3+^ ions are observed at 459 nm, 483 and 578 nm, and 603 nm, respectively. The emission intensity of the Sm^3+^ ions increases with increasing doping concentration of Sm^3+^ and reaches a maximum when the Sm^3+^ doping amount is 3%. The intensity of all of the other emission peaks decreases with increasing doping concentration of Sm^3+^. The decreasing emission intensity of Tm^3+^ ions is easily explained by the ET from Tm^3+^ to Dy^3+^ and Sm^3+^ ions. The overlap of the emission peak of Dy^3+^ and the excitation peak of Sm^3+^, as shown in Figure S2, enable the transfer the energy from Dy^3+^ ions to Sm^3+^ ions, which might result in decreased emission of Sm^3+^ with increasing concentration of Sm^3+^ ions. To investigate this ET mechanism, we plotted the decay curves as a function of dopant concentration (Figure 5b,c). Under excitation at 360 nm, the emission intensities of the Tm^3+^ and Dy^3+^ decay quickly with increasing Sm^3+^ concentration.

The lifetimes of Tm^3+^ at 459 nm were calculated according to the Equation (S5) [29]; those of Dy^3+^ ions at 487 nm were fitted by a biexponential temporal dependence (Table S2), and the average lifetimes τ are calculated through Equations (S2) and (S3). The average lifetimes of Tm^3+^ and Dy^3+^ are reported in the inset tables in Figure 5b,c, where both are observed to decrease slightly with increasing Sm^3+^ concentration. On the basis of ET of the NGT: 3%Tm^3+^/5%Dy^3+^ sample, the ET efficiencies are further enhanced by more than 20%, when the Sm^3+^ ions are co-doped with Tm^3+^ and Dy^3+^. These results confirm the ET from Tm^3+^ to Dy^3+^ and Sm^3+^ and the ET from Dy^3+^ to Sm^3+^ in the NGT host under excitation of Tm^3+^ at a wavelength of 360 nm.

**Figure 6:**
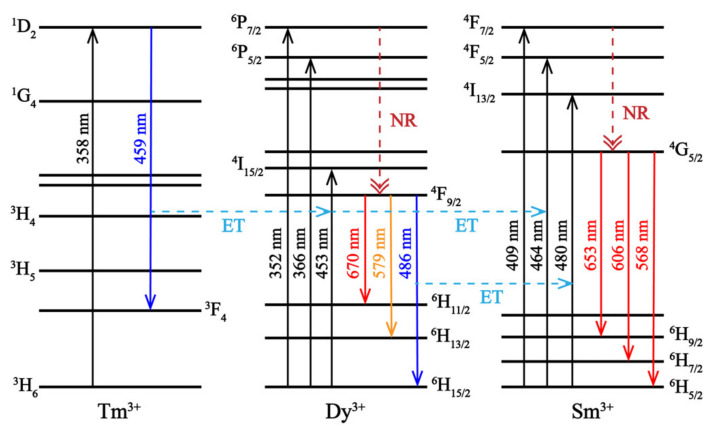
Energy-level diagram for the energy transfer from Tm^3+^ to Dy^3+^ and Sm^3+^ ions and energy transfer from Dy^3+^ to Sm^3+^ ions in the NGT: Tm^3+^/Dy^3+^/Sm^3+^ phosphors. (ET: energy transfer, NR: nonradiative).

The process for the ET of NGT: Tm^3+^/Dy^3+^/Sm^3+^ phosphors can be described in detail using the energy-level diagram in Figure 6. When the phosphors are excited by UV light at 358 nm, energy is absorbed by the Tm^3+^ ions, which emit blue-light with a peak at 459 nm. At the same time, the relaxed energy could also be absorbed by Dy^3+^ and Sm^3+^ ions, thus achieving ET from Tm^3+^ to Dy^3+^ and Sm^3+^ and resulting in blue, yellow, and red-emission peaks. When the electrons fall to the ground state from the excited states of Dy^3+^, energy can also be transferred to the Sm^3+^ ion, resulting in red-emission. The blue, yellow, and red-emissions combine, resulting in white-light.

We obtained the CIE chromaticity diagram for the NGT: 3%Tm^3+^/5%Dy^3+^/*h*%Sm^3+^ phosphors (Figure 3a). The chromaticity coordinates were tunable from blue-, red-, and yellow-light regions to the white-light region. The chromaticity coordinates of NGT: 3%Tm^3+^/5%Dy^3+^/2%Sm^3+^ sample were calculated to be (0.2767, 0.2536), which are near the equal-energy point (0.3333, 0.3333) and comparable with similar results in in previous literatures (Figure S3 and Table S3). Meanwhile, the QY for the phosphors NGT: 3%Tm^3+^/5%Dy^3+^ and NGT: 3%Tm^3+^/5%Dy^3+^/2%Sm^3+^ were measured to be 0.25 and 0.21, respectively. These results indicate that the NGT: Tm^3+^/Dy^3+^/Sm^3+^ phosphor is suitable for commercial applications.

## 4. Conclusions

A series of Tm^3+^-, Dy^3+^-, Sm^3+^-, Tm^3+^/Dy^3+^-, Tm^3+^/Sm^3+^-, and Tm^3+^/Dy^3+^/Sm^3+^-doped NGT phosphors were synthesized via a solid-state reaction method, and their luminescence properties were investigated in detail. The Tm^3+^ single-doped NGT phosphor emits blue-light at 459 nm under excitation by UV light. The Dy^3+^ single-doped NGT phosphor emits blue-light at 486 nm and yellow-light at 579 nm under excitation by UV light, showing white-light emission. The single-doped Sm^3+^ phosphor shows red-light emission under excitation by 409 nm near-UV light. When Dy^3+^ and Sm^3+^ ions are co-doped into the phosphor NGT: 3%Tm^3+^, color-tunable emission is achieved, with chromaticity coordinates changing from (0.1673, 0.0953) to (0.2468, 0.2359) and (0.2891, 0.2171), respectively. White-light with chromaticity coordinates of (0.2767, 0.2536) was finally obtained by doping Sm^3+^ ions into the NGT: 3%Tm^3+^/5%Dy^3+^ phosphor. The results are may be the simultaneous ET from Tm^3+^ to Dy^3+^ and Sm^3+^ ions and ET from Dy^3+^ to Sm^3+^ ions.

## Supplementary Materials

The following are available online at https://www.mdpi.com/2079-4991/10/7/1249/s1. Figure S1: (a, b) The PLE and PL spectra of NGT: *y*%Dy^3+^(*y* = 1, 3, 5, 7, 9) phosphors; (c) the variation of the emission intensity with respect to the concentration of Dy^3+^ ions; (d-e) The PLE and PL spectra of NGT: *x*%Tm^3+^(*x* = 1, 2, 3, 5, 7) phosphors; (f) variation of the emission intensity with respect to the concentration of Tm^3+^ ions; (g-h) PLE and PL spectra of phosphors NGT: *z*%Sm^3+^ (*z* = 1, 2, 3, 4, 5); (i) variation of the emission intensity with respect to the concentration of Sm^3+^ ions; Figure S2: The overlap between PL emission spectrum of NGT: Dy^3+^ phosphor and PL excitation spectra of NGT: Sm^3+^; Figure S3: Comparison of the CIE chromaticity diagram of the WLED phosphors in previous literatures; Table S1: CIE coordinates of the as-prepared phosphors; Table S2: Fitting parameters of the PL decay curves; Table S3: Comparison of the CIE coordinates of the WLED phosphors in previous literatures.

## References

[1] Feldmann C., Jüstel T., Ronda C.R., Schmidt P.J. (2003). Inorganic Luminescent Materials: 100 Years of Research and Application. Adv. Funct. Mater..

[2] Lee S.-W., Seo J.M., Lee M.-K., Chun J.-H., Antonisamy P., Arasu M.V., Suzuki T., Al-Dhabi N.A., Kim S.-J. (2014). Influence of different LED lamps on the production of phenolic compounds in common and Tartary buckwheat sprouts. Ind. Crops Prod..

[3] Rodríguez-Vidal E., Otaduy D., Ortiz D., González F., Moreno F., Saiz J.M. (2014). Optical performance of a versatile illumination system for high divergence LED sources. Optik.

[4] Rosemann N.W., Eußner J.P., Beyer A., Koch S.W., Volz K., Dehnen S., Chatterjee S. (2016). A highly efficient directional molecular white-light emitter driven by a continuous-wave laser diode. Science.

[5] Nishida T., Ban T., Kobayashi N. (2003). High-color-rendering light sources consisting of a 350-nm ultraviolet light-emitting diode and three-basal-color phosphors. Appl. Phys. Lett..

[6] Kim J.S., Jeon P.E., Park Y.H., Choi J.C., Park H.L., Kim G.C., Kim T.W. (2004). White-light generation through ultraviolet-emitting diode and white-emitting phosphor. Appl. Phys. Lett..

[7] Kang Y.C., Lenggoro I.W., Park S.B., Okuyama K. (2000). YAG:Ce phosphor particles prepared by ultrasonic spray pyrolysis. Mater. Res. Bull..

[8] Pan Y., Wu M., Su Q. (2004). Comparative investigation on synthesis and photoluminescence of YAG:Ce phosphor. Mater. Sci. Eng. B.

[9] Pan Y., Wu M., Su Q. (2004). Tailored photoluminescence of YAG:Ce phosphor through various methods. J. Phys. Chem. Solids.

[10] Narukawa Y., Niki I., Izuno K., Yamada M., Murazaki Y., Mukai T. (2002). Phosphor-conversion white light emitting diode using InGaN near-ultraviolet chip. Jpn. J. Appl. Phys. Part 2-Lett. Express Lett..

[11] Sheu J.K., Chang S.J., Kuo C.H., Su Y.K., Wu L.W., Lin Y.C., Lai W.C., Tsai J.M., Chi G.C., Wu R.K. (2003). White-light emission from near UV InGaN-GaN LED chip precoated with blue/green/red phosphors. IEEE Photonics Technol. Lett..

[12] Huang C.-H., Chan T.-S., Liu W.-R., Wang D.-Y., Chiu Y.-C., Yeh Y.-T., Chen T.-M. (2012). Crystal structure of blue–white–yellow color-tunable Ca 4 Si 2 O 7 F 2: Eu 2+, Mn 2+ phosphor and investigation of color tunability through energy transfer for single-phase white-light near-ultraviolet LEDs. J. Mater. Chem..

[13] Li L., Zi W., Li G., Lan S., Ji G., Gan S., Zou H., Xu X. (2012). Hydrothermal synthesis and luminescent properties of NaLa(MoO4)2:Dy3+ phosphor. J. Solid State Chem..

[14] Zhong H., Li X., Shen R., Zhang J., Sun J., Zhong H., Cheng L., Tian Y., Chen B. (2012). Spectral and thermal properties of Dy3+-doped NaGdTiO4 phosphors. J. Alloys Compd..

[15] Min X., Fang M., Huang Z., Liu Y., Tang C., Wu X. (2014). Luminescent properties of white-light-emitting phosphor LaMgAl11O19:Dy3+. Mater. Lett..

[16] Min X., Fang M., Huang Z., Liu Y.G., Tang C., Wu X., Dunn B. (2015). Luminescence Properties and Energy-Transfer Behavior of a Novel and Color-Tunable LaMgAl11O19:Tm3+, Dy3+Phosphor for White Light-Emitting Diodes. J. Am. Ceram. Soc..

[17] Li Y., Wang X., Liu W., Wang C., Wang Y. (2016). Photoluminescence properties and energy transfer of a color tunable phosphor: Dy 3+ and Tm 3+ co-activated SrCaAl 2 SiO 7. Mater. Res. Bull..

[18] Xia Z., Chen D. (2010). Synthesis and Luminescence Properties of BaMoO4:Sm3+Phosphors. J. Am. Ceram. Soc..

[19] Samantaray C. (2004). Photoluminescence properties of Eu3+-doped barium strontium titanate (Ba, Sr) TiO3 ceramics. Mater. Lett..

[20] Hu G., Hu X., Chen W., Cheng Y., Liu Z., Zhang Y., Liang X., Xiang W. (2017). Luminescence properties and thermal stability of red phosphor Mg2TiO4:Mn4+ additional Zn2+ sensitization for warm W-LEDs. Mater. Res. Bull..

[21] Thomas K., Alexander D., Sisira S., Biju P.R., Unnikrishnan N.V., Ittyachen M.A., Joseph C. (2017). NUV/blue LED excitable intense green emitting terbium doped lanthanum molybdate nanophosphors for white LED applications. J. Mater. Sci. Mater. Electron..

[22] Lv L., Wang S., Wang X., Han L. (2018). Inducing luminescent properties of Mn4+ in magnesium titanate systems: An experimental and theoretical approach. J. Alloys Compd..

[23] Talewar R.A., Mahamuda S., Swapna K., Rao A.S. (2019). Near UV based Dy3+ ions doped alkaline-earth chloro borate glasses for white LED’s and visible lasers. Optics Laser Technol..

[24] Trápala-Ramírez A.U., Gálvez-Sandoval J.L.N., Lira A., Camarillo I., Alvarez-Ramos E., Meza-Rocha A.N., Caldiño U. (2019). Calcium-zinc phosphate glasses activated with Tb3+/Eu3+ for laser and white LED applications. J. Lumin..

[25] Byeon S.H., Park K., Itoh M. (1996). Structure and Ionic Conductivity of NaLnTiO4; Comparison with Those of Na2Ln2Ti3O10 (Ln= La, Nd, Sm, and Gd). J. Solid State Chem..

[26] Zhou A., Song F., Song F., Feng M., Adnan K., Ju D., Wang X. (2018). Optical thermometry using fluorescence intensities multi-ratios in NaGdTiO4:Yb3+/Tm3+ phosphors. Opt. Mater..

[27] Li X., Chen B., Shen R., Zhong H., Cheng L., Sun J., Zhang J., Zhong H., Tian Y., Du G. (2011). Fluorescence quenching of 5DJ (J= 1, 2 and 3) levels and Judd–Ofelt analysis of Eu3+ in NaGdTiO4 phosphors. J. Phys. D Appl. Phys..

[28] Li X., Wang X., Zhong H., Cheng L., Xu S., Sun J., Zhang J., Li X., Tong L., Chen B. (2016). Effects of Er3+ concentration on down-/up-conversion luminescence and temperature sensing properties in NaGdTiO4: Er3+/Yb3+ phosphors. Ceram. Int..

[29] Bharat L.K., Du P., Yu J.S. (2016). Long-wave UVA radiation excited warm white-light emitting NaGdTiO4: Tm3+/Dy3+/Eu3+ ions tri-doped phosphors: Synthesis, energy transfer and color tunable properties. J. Alloys Compd..

[30] Ahrens L.H. (1952). The Use of Ionization Potentials Part 1. Ionic Radii of the Elements. Geochim. Cosmochim. Acta.

[31] Van Uitert L.G. (1967). Characterization of Energy Transfer Interactions between Rare Earth Ions. J. Electrochem. Soc..

[32] Chung J.H., Lee S.Y., Shim K.B., Kweon S.-Y., Ur S.-C., Ryu J.H. (2012). Blue upconversion luminescence of CaMoO4:Li+/Yb3+/Tm3+ phosphors prepared by complex citrate method. Appl. Phys. A.

[33] Fan B., Liu J., Zhao W., Han L. (2020). Luminescence properties of Sm3+ and Dy3+ co-doped BaY2ZnO5 phosphor for white LED. J. Lumin..

[34] Lakshminarayana G., Yang H., Qiu J. (2009). White light emission from Tm3+/Dy3+ co-doped oxyfluoride germanate glasses under UV light excitation. J. Solid State Chem..

[35] Lv C., Min X., Li S., Huang Z., Liu Y.G., Wu X., Fang M. (2018). Luminescence properties of emission tunable single-phased phosphor La 7 O 6 (BO 3)(PO 4) 2: Ce 3+, Tb 3+, Eu 3+. Mater. Res. Bull..

