# Supplementary material for: Multiple Energy Transfer in Luminescence-Tunable Single-Phased Phosphor NaGdTiO4: Tm3+, Dy3+, Sm3+
Source: Nanomaterials (Basel). 2020 Jun 27;10(7):1249. doi: 10.3390/nano10071249 (PMC7407989; doi:10.3390/nano10071249)
Supplement: Supplementary file 1 [file nanomaterials-10-01249-s001.pdf]

Support Information

Multiple energy transfer in luminescenc-tunable single-phased phosphor $NaGdTiO_4$: Tm^{3+}, Dy^{3+}, Sm^{3+}

Jun Xiao, Cong Wang, Xin Min*, Xiaowen Wu, Yangai Liu, Zhaohui Huang and Minghao Fang

Beijing Key Laboratory of Materials Utilization of Nonmetallic Minerals and Solid Wastes, National Laboratory of Mineral Materials, School of Materials Science and Technology, China University of Geosciences (Beijing), Beijing 100083, China

**Corresponding author Tel: +86-010-82322186, Fax: +86-010-82322186*

E-mail: minx@cugb.edu.cn

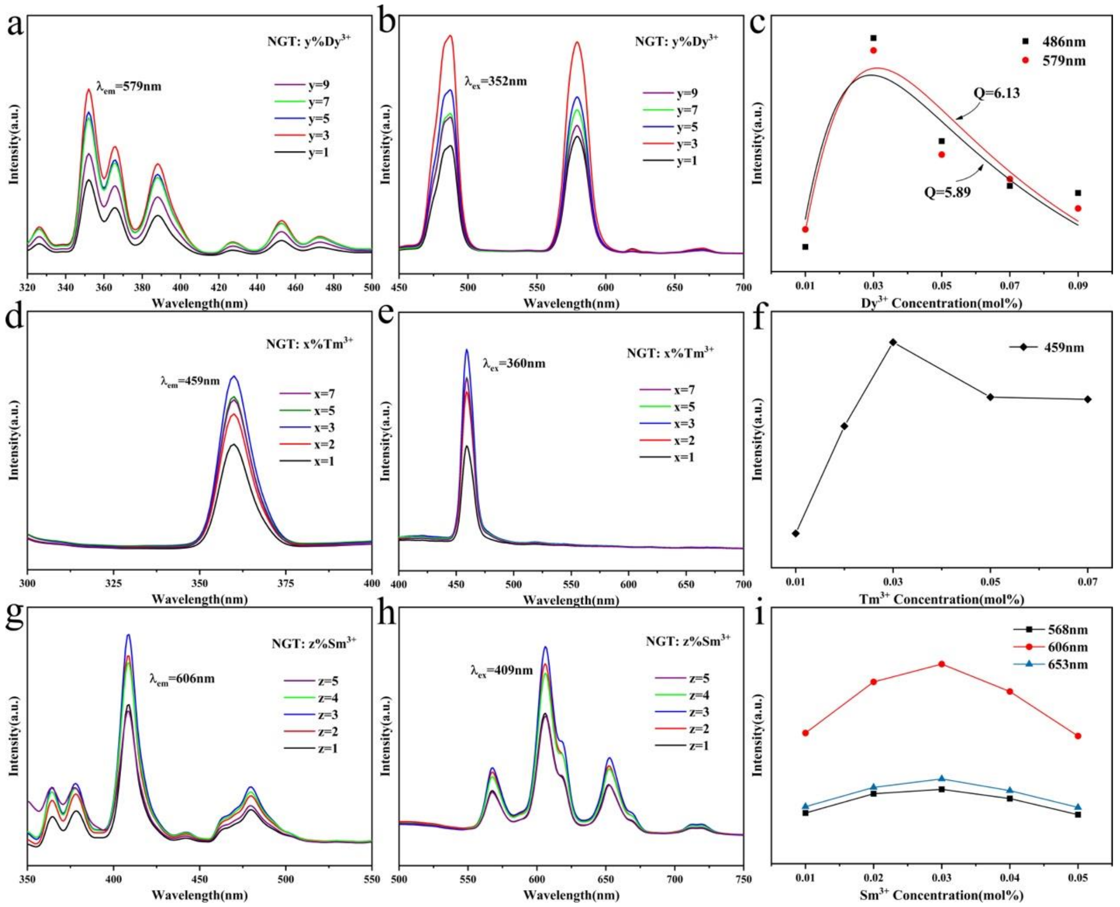

Figure S1. (a, b) The PLE and PL spectra of NGT: y%Dy^{3+}(y = 1, 3, 5, 7, 9) phosphors; (c) the variation of the emission intensity with respect to the concentration of Dy^{3+} ions; (d-e) The PLE and PL spectra of NGT: x%Tm^{3+}(x = 1, 2, 3, 5, 7) phosphors; (f) variation of the emission intensity with respect to the concentration of Tm^{3+} ions; (g-h) PLE and PL spectra of phosphors NGT: z%Sm^{3+} (z = 1, 2, 3, 4, 5); (i) variation of the emission intensity with respect to the concentration of Sm^{3+} ions.

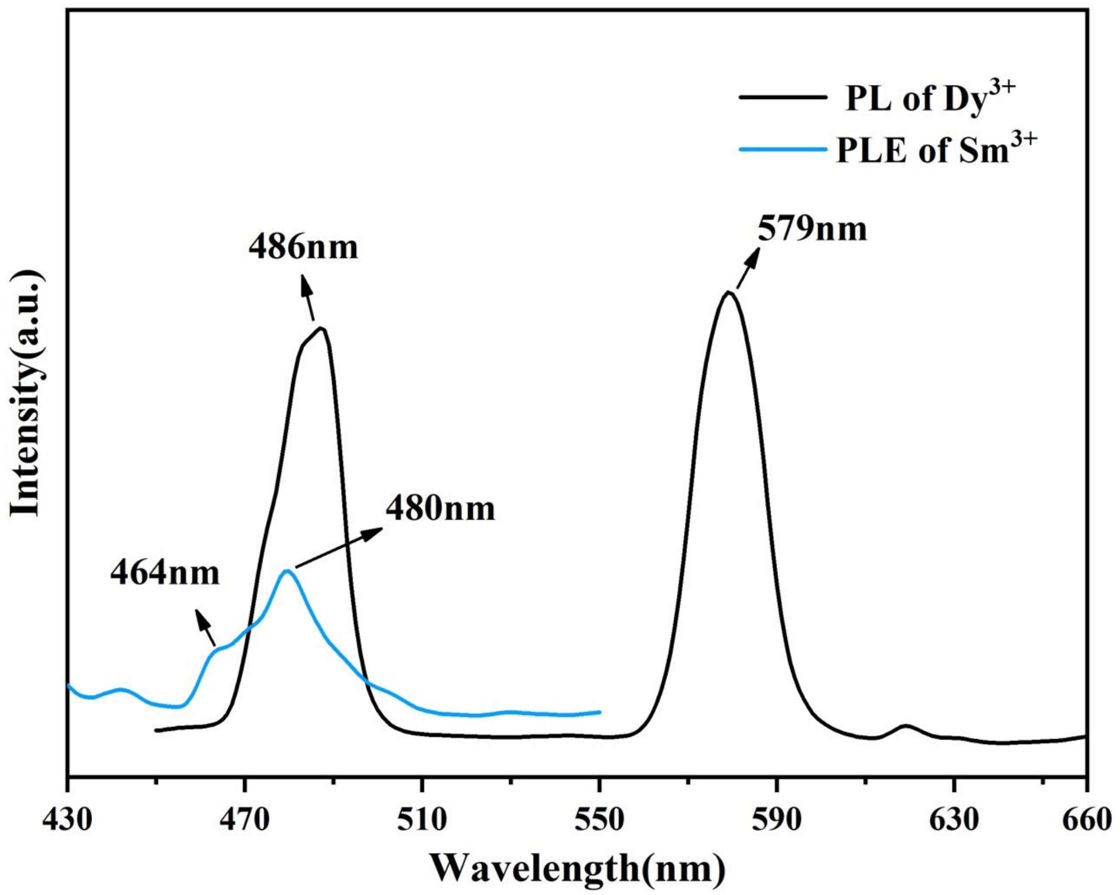

Figure S2. The overlap between PL emission spectrum of NGT: Dy^{3+} phosphor and PL excitation spectra of NGT: Sm^{3+}.

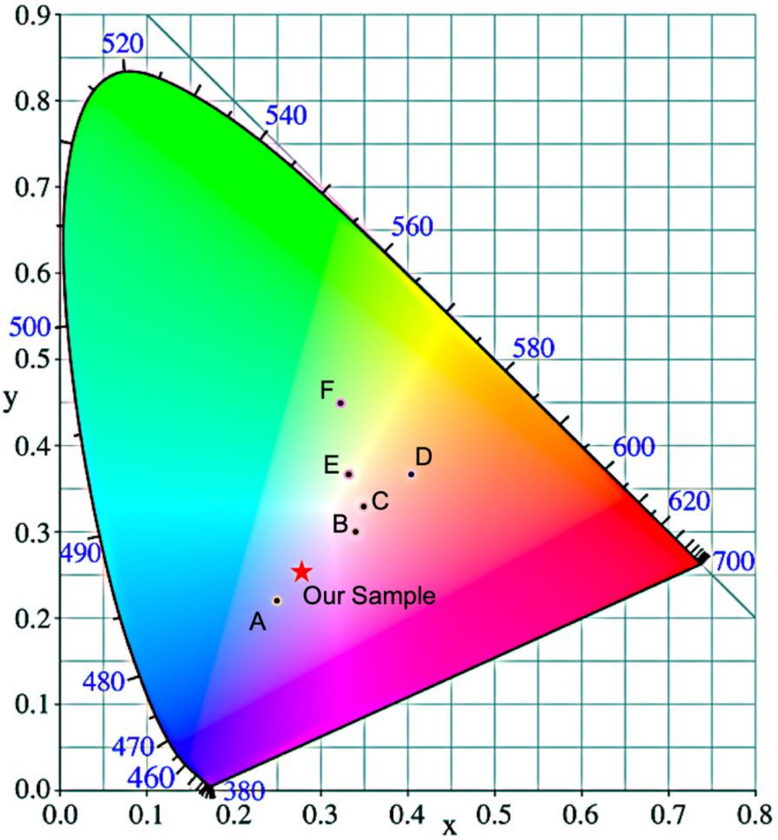

Figure S3. Comparison of the CIE chromaticity diagram of the WLED phosphors in previous literatures.

$$\mathrm{I} = \frac{\mathrm{C}}{\mathrm{k}\left(1+\beta \mathrm{C}^{Q/3}\right)} \qquad \text{Eq. S1}$$

In this formula, I represents the luminescence intensity of the as-prepared samples, C is the doping concentration of the activator ions, k and β are constants, and Q represents the interaction between the rare-earth ions.

$$I_t = I_0 + A_1 \cdot \exp(-t/\tau_1) + A_2 \cdot \exp(-t/\tau_2) \qquad \text{Eq. S2}$$

$$\tau = (\tau_1^2 A_1 + \tau_2^2 A_2)/(\tau_1 A_1 + \tau_2 A_2) \qquad \text{Eq. S3}$$

where τ_1 and τ_2 are the decay times of different components with intensities A_1 and A_2, respectively.

$$\eta = 1 - {}^{\tau_s}/_{\tau_0} \qquad \text{Eq. S4}$$

where η is the energy transfer efficiencies, τ_s and τ_0 are the decay time.

$$I_t = I_0 + A \cdot \exp(-t/\tau) \qquad \text{Eq. S5}$$

where I_t is the luminescence intensity at the time t and I_0 and A are the constants.

Table S1. CIE coordinates of the as-prepared phosphors.

Number	Samples	Excitation wavelength (nm)	CIE coordinates	
			x	y
A1	NGT: 1%Dy^{3+}	352	0.3664	0.3881
A2	NGT: 3%Dy^{3+}		0.3544	0.3794
A3	NGT: 7%Dy^{3+}		0.3504	0.3783
A4	NGT: 9%Dy^{3+}		0.3471	0.3770
B1	NGT: 1%Tm^{3+}	360	0.1773	0.1164
B2	NGT: 2%Tm^{3+}		0.1698	0.1028
B3	NGT: 3%Tm^{3+}		0.1673	0.0953
C1	NGT: 1%Sm^{3+}	409	0.5537	0.4351
C2	NGT: 2%Sm^{3+}		0.5638	0.4270
C3	NGT: 3%Sm^{3+}		0.5737	0.4189
D1	NGT: 3%Tm^{3+}/1%Dy^{3+}	360	0.2029	0.1673
D2	NGT: 3%Tm^{3+}/2%Dy^{3+}		0.2315	0.2114
D3	NGT: 3%Tm^{3+}/3%Dy^{3+}		0.2468	0.2359
E1	NGT: 3%Tm^{3+}/1%Sm^{3+}	360	0.2204	0.1518
E2	NGT: 3%Tm^{3+}/2%Sm^{3+}		0.2363	0.1708
E3	NGT: 3%Tm^{3+}/4%Sm^{3+}		0.2891	0.2171
F	NGT: 3%Tm^{3+}/5%Dy^{3+}/2%Sm^{3+}	360	0.2767	0.2536

Table S2. Fitting parameters of the PL decay curves.

Sample	I_0	A_1	τ_1(μs)	A_2	τ_2(μs)
NGT: 3%Tm^{3+}/0%Dy^{3+}	1.367	1.375	8.229	1.084	390.0
NGT: 3%Tm^{3+}/1%Dy^{3+}	0.701	1.024	2.171	1.817	239.0
NGT: 3%Tm^{3+}/3%Dy^{3+}	0.990	1.036	3.242	1.553	192.5
NGT: 3%Tm^{3+}/5%Dy^{3+}	1.065	1.150	2.780	1.347	182.8
NGT: 3%Tm^{3+}/7%Dy^{3+}	1.028	1.809	2.644	1.072	138.1
NGT: 3%Tm^{3+}/0%Sm^{3+}	1.367	1.375	8.229	1.084	390.0
NGT: 3%Tm^{3+}/1%Sm^{3+}	1.118	1.321	8.131	1.120	371.1
NGT: 3%Tm^{3+}/2%Sm^{3+}	0.991	1.272	10.227	1.149	343.4
NGT: 3%Tm^{3+}/4%Sm^{3+}	1.107	1.409	8.181	1.069	253.7
NGT: 3%Tm^{3+}/5%Dy^{3+}/1%Sm^{3+}	1.271	109.175	10.786	1.075	320.0
NGT: 3%Tm^{3+}/5%Dy^{3+}/2%Sm^{3+}	1.163	123.920	10.383	1.069	266.0
NGT: 3%Tm^{3+}/5%Dy^{3+}/3%Sm^{3+}	0.994	144.943	9.971	1.024	349.2

Table S3. Comparison of the CIE coordinates of the WLED phosphors in previous literatures.

	Sample	x	y	Ref.
Our Sample	$NaGdTiO_4$: $0.03Tm^{3+}/0.05Dy^{3+}/0.02Sm^{3+}$	0.2767	0.2536	-
A	YAG: $0.05Ce^{3+}$	0.2498	0.2201	[1]
B	CdSe/ZnS/CdSe	0.34	0.30	[2]
C	$Sr_3MgSi_2O_8$: $0.02Eu^{2+}$, $0.05Mn^{2+}$	0.35	0.33	[3]
D	BaY_2ZnO_5: $0.14Dy^{3+}$, $0.04Sm^{3+}$	0.404	0.367	[4]
E	$LaMgAl_{11}O_{19}$: $0.1Dy^{3+}$	0.3324	0.3665	[5]
F	$Na_3YSi_3O_9$: $0.03Sm^{3+}$, $0.09Tb^{3+}$, $0.02Tm^{3+}$	0.3231	0.4491	[6]

References

1. Hu, S.; Lu, C.; Zhou, G.; Liu, X.; Qin, X.; Liu, G.; Wang, S.; Xu, Z. Transparent YAG:Ce ceramics for WLEDs with high CRI: Ce^{3+} concentration and sample thickness effects. *Ceram. Int.* **2016**, *42*, 6935-6941, doi:10.1016/j.ceramint.2016.01.079.
2. Nizamoglu, S.; Mutlugun, E.; Ozel, T.; Demir, H.V.; Eychmuller, A. Multi-layered CdSe/ZnS/CdSe heteronanocrystals to generate and tune white light. *Conference Proceedings Lasers & Electro Optics Society Annual Meeting Leos* **2008**.
3. Kim, J.S.; Jeon, P.E.; Park, Y.H.; Choi, J.C.; Park, H.L.; Kim, G.C.; Kim, T.W. White-light generation through ultraviolet-emitting diode and white-emitting phosphor. *Appl. Phys. Lett.* **2004**, *85*, 3696-3698, doi:10.1063/1.1808501.
4. Fan, B.; Liu, J.; Zhao, W.; Han, L. Luminescence properties of Sm^{3+} and Dy^{3+} co-doped BaY_2ZnO_5 phosphor for white LED. *J. Lumin.* **2020**, *219*, doi:10.1016/j.jlumin.2019.116887.
5. Min, X.; Fang, M.; Huang, Z.; Liu, Y.; Tang, C.; Wu, X. Luminescent properties of white-light-emitting phosphor $LaMgAl_{11}O_{19}:Dy^{3+}$. *Mater. Lett.* **2014**, *125*, 140-142, doi:10.1016/j.matlet.2014.03.171.
6. Zhao, W.; An, S.; Fan, B.; Li, S. Luminescence properties of $Na_3YSi_3O_9:M^{3+}$ (M = Sm, Tb, Tm) glass ceramics. *J. Alloys Compd.* **2013**, *566*, 142-146, doi:10.1016/j.jallcom.2013.02.166.
